# Modeling Multi-Agent Self-Organization through the Lens of Higher Order Attractor Dynamics

**DOI:** 10.3389/fpsyg.2017.00380

**Published:** 2017-03-20

**Authors:** Jonathan E. Butner, Travis J. Wiltshire, A. K. Munion

**Affiliations:** Department of Psychology, University of UtahSalt Lake City, USA

**Keywords:** dynamical systems, social coordination dynamics, multi-agent coordination, attractors, agent-based modeling

## Abstract

Social interaction occurs across many time scales and varying numbers of agents; from one-on-one to large-scale coordination in organizations, crowds, cities, and colonies. These contexts, are characterized by emergent self-organization that implies higher order coordinated patterns occurring over time that are not due to the actions of any particular agents, but rather due to the collective ordering that occurs from the interactions of the agents. Extant research to understand these social coordination dynamics (SCD) has primarily examined dyadic contexts performing rhythmic tasks. To advance this area of study, we elaborate on attractor dynamics, our ability to depict them visually, and quantitatively model them. Primarily, we combine difference/differential equation modeling with mixture modeling as a way to infer the underlying topological features of the data, which can be described in terms of attractor dynamic patterns. The advantage of this approach is that we are able to quantify the self-organized dynamics that agents exhibit, link these dynamics back to activity from individual agents, and relate it to other variables central to understanding the coordinative functionality of a system's behavior. We present four examples that differ in the number of variables used to depict the attractor dynamics (1, 2, and 6) and range from simulated to non-simulated data sources. We demonstrate that this is a flexible method that advances scientific study of SCD in a variety of multi-agent systems.

## Introduction

For many animals and humans, social interaction is pervasive in daily life. Social interaction occurs across many time scales and varying numbers of agents; from one-on-one to large-scale coordination in organizations, crowds, cities, and colonies. Since social interactions occur at different scales, and in ways that change dynamically over time, they can be quite a complex phenomenon to study without appropriate guiding theoretical and methodological frameworks.

In dynamical systems theory, complexity arguably occurs due to the emergent, self-organizational nature of the system. Emergent, self-organization here implies that there are higher order macroscopic patterns occurring over time that are not necessarily due to the actions of any particular controlling agents or components, but rather due to the collective ordering that occurs from the individual interactions of the agents or components of the system (Halley and Winkler, 2008). Taken together these diffuse interactions contribute to more macro-scale phenomena that are observed over time. Common examples of this type of emergent, self-organization of social behavior occurs in flocking birds and in schools of fish that appear to move in a highly coordinated fashion (e.g., Couzin and Krause, 2003).

Because of the multitude of agents (or system components) that give rise to emergent patterns, it is often difficult to determine how one should depict the resultant system. In line with approaches to *social coordination dynamics* (SCD), we aim to uncover the dynamic processes that underlie the ways in which agents are able to organize their behavior and change together in time (Oullier and Kelso, 2009). This emergence is a form of c*oordination* that specifically implies the occurrence of a functional ordering of components that interact across spatial and temporal dimensions, often with multi-directional relationships (Kelso, 2009; Butner et al., 2014a). We aim to model this emergent, multi-agent coordination through attractor dynamics depictions (which we discuss in detail in the next section).

From the SCD perspective, two or more agents are able to coordinate their behavior based on some form of mutual information exchange. This information exchange generates coordinative structures with higher order patterns not easily identifiable from the lower order interactions. The resultant higher order patterns are then depicted through attractor dynamics in which the patterns are attributed with stability properties implied by an underlying topology (Kelso, 1995). SCD is thus consistent with notions of weak emergence (Bedau and Humphreys, 2008), but the resultant patterns have then been modeled using attractor dynamics descriptions depicting patterning over time that have stable properties under perturbations. One major distinction between SCD and examples of weak emergence (usually through agent-based or cellular automata models) is in the scale of the social systems involved.

Agent-based models are usually quite large-scale social systems, while SCD has often focused on a dyadic scale of analysis. SCD has excelled in generating models of intentional and spontaneous dyadic interpersonal rhythmic behavior such as finger or limb oscillations (e.g., Haken et al., 1985; Schmidt et al., 1990; Oullier et al., 2008), swinging pendula (Schmidt and O'Brien, 1997), and rocking in chairs (Richardson et al., 2007). Some recent work has provided ways to assess social interactions in larger scales such as coordination of groups bigger than dyads (e.g., Richardson et al., 2012; Duarte et al., 2013). One challenge is to generate models of emergent, multi-agent coordination in social systems where the agents may not behave rhythmically *per se*, but are following some organizing rules or structures that give rise to coordinated behavior serving a functional purpose.

In the current paper we build on SCD approaches, by modeling the results from large-scale agent-based systems as a function of attractor dynamics. Our chosen technique utilizes mixture modeling in conjunction with topological equations to represent attractor dynamics. This approach is particularly attractive in that topological representations of phase space can yield more qualitative information in comparison to other time series approaches (Strogatz, 2014), generating a more complete picture of the underlying system dynamics. Specifically, we examine a series of agent-based examples, and model each set of time series as a function of their changes through time. We show how sets of linear equations can depict the higher order emergent patterns in ways consistent with attractor dynamics. The advantage of this approach is that we are able to quantify the self-organized dynamics that agents exhibit, link these dynamics back to activity from individual agents, and relate it to other variables central to understanding the coordinative functionality of a system's behavior. Our goal is to exemplify the strategy. In all, we present four examples that differ in the number of variables used to depict the attractor dynamics (i.e., the dimensionality of the systems) and range from simulated to non-simulated data sources.

### Attractor dynamics

In dynamical systems theory, the concern is often placed on what states a system is drawn toward, or away from, as it changes over time (e.g., Richardson et al., 2014). This epitomizes the notion of attractor dynamics. *Attractor dynamics* are merely a mathematical way of expressing repetitious behavior in the face of constant disruptions to those repetitions. The constant disruptions are inherently part of the system in that open systems are dissipative and function far from equilibrium to maintain patterns (Prigogene and Stangers, 1984). By only examining a portion of a system, as is common in empirical research, the unexamined variables are treated as constant disruptions or perturbations to those patterns. These repetitious behaviors describe the most probable system states and their ability to remain in these states (while facing perturbations) conveys the inherent stability of those states. These attractor dynamics can then be modeled using differential/difference equations, allowing for exploration of the inferred dynamics and theorizing the manifolds in which the system functions (Differential equations are based on idealized models for when change in time approaches zero while difference equations estimate models using the observed discrete differences; Butner et al., 2015).

Assuming a system exhibits stability, the emergence of a limited set of patterns, which can be described in terms of topological features, are plausible. These topological features can be described using map analogies, because there is a strong tie between topology and maps. In fact, differential topology *is* the math behind maps. Traditionally, topographical maps convey elevation of a landscape. But, the notion of topologies can also be applied as a graphical representation of how data are changing over time.

To ease the interpretation of differential topology, we will temporarily link movement on maps to different topological features. This interpretation is directly relevant to several of the examples (although more general definitions are extant; Butner et al., 2015). An *Attractor* is when the agents are drawn toward a particular coordinate over time or a particular directional heading. This is akin to a topographical valley. A *Repeller* is a coordinate that agents move away from. These would be reflected topographically as a mountain peak. A *Saddle* occurs when agents are attracted in one dimension and repelled in another. It is analogous to a topographical ridgeline because it can separate different patterns such as two attractors (Abraham and Shaw, 1983; Butner et al., 2015). A *Cycle* corresponds to a push/pull of two dimensions on one another. Combined versions of patterns described can also be observed such as spiral attractors where there are circling movements for how agents converge toward an attractor. Saddles and cycles require at least two dimensions and thus will only be possible in the later examples (not merely with heading as it is a one dimensional example).

To continue with the link to maps, we will begin with agent-based models that function spatially. As a simplification, we can reduce their behavior to movement along an X and Y axis or merely the directional heading of agents (when we only require a single dimension to depict the system). We can then model the simultaneous change of these variables over time. In this way, we capture the movement of many agents and can characterize them with attractor descriptions. With this information it is possible to examine and identify patterns of change for the overall system using the particular topological features defined above to describe how multiple agents are moving over time (Butner et al., 2015). It is in these terms that we gain an understanding of the emergent, coordination of many agents.

**Figure 1:**
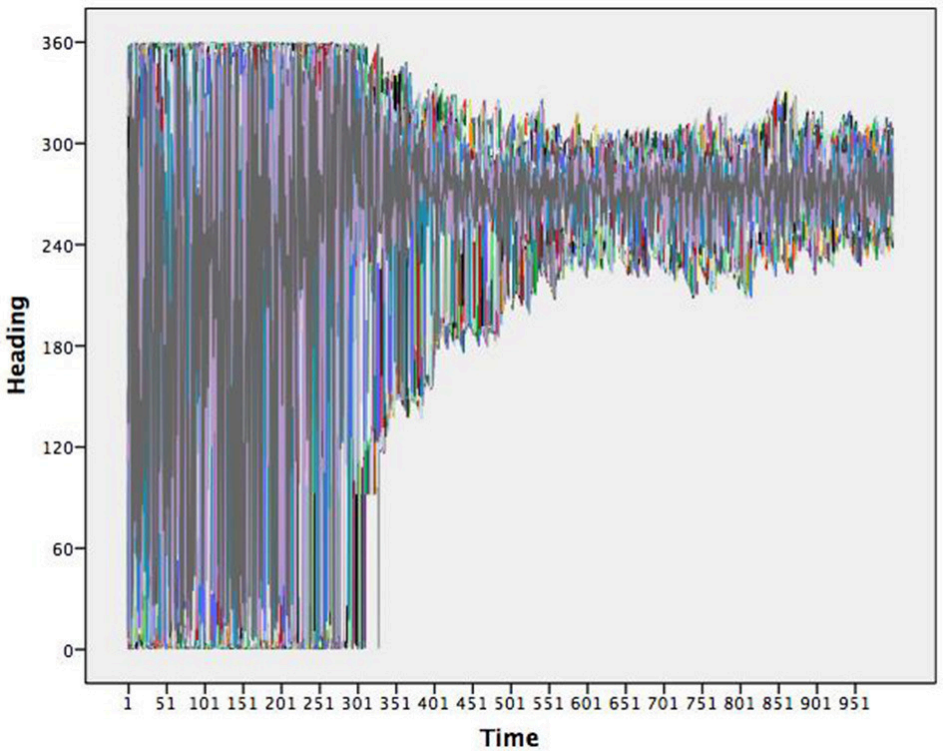
**Time series of the headings for 300 flocking agents**. Note that the flock moves toward a very restricted heading.

As a beginning example, consider a Flocking agent based model (Wilensky, 1998) in NetLogo v5.2.1 (Wilensky, 1999) designed to emulate the self-organized behavior of how flocks of birds might come to match one another's movements creating complex group behavior. Agents start with a random heading and constant velocity in a wrapped environment (makes a torus). The heading for each agent is determined by three rules: (1) *alignment* states that each agent tends to turn to be moving in the same direction as nearby agents; (2) *separation* states that each agent will turn to avoid an agent when it gets too close; and (3) *cohesion* states that agents tend to move toward other agents. As the agents “fly” through the two dimensional environment they update their headings over time. Figure 1 shows time series of the headings for all (300) agents simultaneously over one thousand iterations. It is clear that early in the simulation the full range of headings are observed yet, in later times the range of headings become more restricted and shared by the agents. This is an example of the emergent coordination that occurs within the Flocking model.

To depict these results topologically requires identifying the underlying map in which the agents are interacting. An attractor, in this case, would be the heading(s) in which the agents move toward and the stability would be the resistance exhibited in the system when an agent begins to diverge from this attractive heading and pulled back thusly. The map is not one of actual hills and valleys, but instead the resultant decrease in heading directions, from the emergent self-organization between agents. Thus, the trajectories for agents imply an underlying pattern that we can infer. One assumption of dynamical systems theory is that there is one—or perhaps multiple—underlying patterns emergent from the interactions of individual agents over time. Interactions result in a consistent pattern, that the system flexibly returns to when interactions or outside forces briefly move the system away from its primary pattern. This notion of consistency in the face of perturbations is stability. While it is easy to observe the convergence of heading amongst the agents in Figure 1, little information can be drawn in regards to the number of underlying patterns and their inherent stabilities.

The flocking example is a useful one in that the implied map is not a map of X and Y coordinates, but one of heading—it is a one dimensional map. One dimensional maps are not very interesting to draw; they are a line showing where the data converges over time. That is, attractor dynamics are time implicit models rather than time explicit ones and thus, are akin to collapsing the X axis in Figure 1, while adding in notions of *where each agent goes next* to determine the map.

Dynamical systems theory has long provided the theoretical framework and terminology for describing multi-agent self-organized patterning. Returning to Figure 1, an apt depiction of the Flocking simulation is one that begins with many attractors that cease to exist over time, which produce a limited set of stable attractors. This qualitative description captures the evolving process, without any of the quantitative dynamics. By quantifying them through topological equation representations, we can further differentiate aspects of the system and specify the strength of the attractors. We therefore next cover the step of quantifying the dynamic.

#### A vector based approach

There are several ways to estimate differential topological equations. In all cases, we must first express the data in terms of data vectors rather than values. For the heading data illustrated in Figure 1, the data is structured such that two or more points in time are used to define a data vector, known as a time delay or Toeplitz data structure (e.g., Boker and Laurenceau, 2006). The data is structured so that a value at time t and a value at time *t*+1 are two variables within the model. Further, our models are all estimated in structural equation modeling wherein change was built into the models themselves as latent variables (McArdle, 2009). One can also estimate change directly through a discrete difference or various methods for estimating derivatives and thus while we use structural equations to build our models, this is far from a necessity (Boker et al., 2010).

Different attractor dynamics are then captured through expressions of change predicted by value. For example, Equation (1) expresses the potential dynamics for the headings of the various agents.

(1) $$\begin{array}{r} {ẋ}_{t}=b_{0}+b_{1}x_{t}+e_{t} \end{array}$$

Current heading of a given agent at time *t* is *x, x*-dot is an estimate of its derivative with respect to time, *b*_0_ is the intercept, *b*_1_, the slope with respect to *x* and *e*_*t*_ is error. For clarity, this equation is written in regression form where velocity in heading at each point in time is treated as the criterion and position (current heading) is the predictor. When the slope in Equation 1 is negative, we observe an attractor where the time series are attracted toward a value of −*b*_0_/*b*_1_ known as the set point (Butner et al., 2015). A repeller occurs when the slope is positive instead of negative. The strength of attraction/repulsion is defined by the steepness of the slope relative to zero.

Equation (1) is limited in that it can only capture a single topological feature (Butner et al., 2015). While the system may converge to a single heading, this convergence is developed over time. Consistent with the qualitative description of the flocking model, we should observe several patterns that cease to exist as time continues. This results in a much more limited set of dynamic patterns that occur at later times. We therefore expand our approach to allow for multiple sets of Equation (1). We did this through an analytic technique known as mixture modeling.

#### Mixture modeling methods

Mixture modeling is a taxonomic approach that can be combined with structural equation modeling (Enders, 2006) as an alternative way to capture interactions (Jung and Wickrama, 2008). Non-linear dynamical systems allow for multiple topological patterns by building non-linear transformations, such as the interaction and therefore mixture modeling can be used as a way to capture the different topological features by slicing up the overarching state space under an assumption that each dynamic is locally linear. One description of mixture modeling is as a multiple group analysis (stacked model), where assignment to group is unknown (Muthen, 2001). Multiple group models allow for different parameters across groups. We can extract different equation sets by allowing key parameters to differ across these groups while equating others. Specifically, we allowed the slope coefficients characterizing how position predicted each velocity, the intercepts for the velocity factors, the means for the position factors, the residual variances for the velocity factors, and the variances for the position factors to vary across sets of equations [see Appendix A (Supplementary Material) for an example in Mplus (Muthén and Muthén, 1998–2012)].

As previously described, the sign of the slope coefficients capture the type (e.g., attractor, repeller, limit cycle) and strength of attraction for the dynamic implied by the equations (see also Butner et al., 2015). In addition, the velocity intercepts help determine the set point, or relative position to which the dynamics can be described (e.g., the location of the attractor). Following logic laid out under notions of centering and simple slopes analysis (Cohen et al., 2003), the means and variances for the position factors help depict common trajectories implied by the pattern and thus help identify the basin of attraction. By allowing for variation in these parameters across latent classes, we can infer a number of varying topological features, as opposed to a single feature.

Mixture modeling can be used as a confirmatory or exploratory method. In either case, there must be established criteria for fit. The current preferred methods are through forms of the Bayesian Information Criterion (BIC) or through forms of model testing such as log likelihood or chi-square comparisons to see if the current number of extracted groups improves description of the data beyond the previous number of groups. Specifically, the BIC and sample size adjusted BIC tend to minimize when the proper number of mixture groups has been extracted (Sclove, 1987; Nagin and Tremblay, 1999; Nylund et al., 2007) and both have been used under different circumstances usually relating to the sample size (sample size adjusted BIC is preferred when *n* < Bauer and Curran, 2003; Lubke and Neale, 2006; Enders and Tofighi, 2007).

Model identification can also be informed by various likelihood ratio tests (LRT), which are used to test relative model fit by testing the null hypothesis that competing models demonstrate comparable fit (Vuong, 1989). Within latent variable models such as the present one, the Vuong-Lo-Mendell-Rubin test (Lo et al., 2001) is an accepted methodology for testing the equivalence of two associated probability density functions (Henson et al., 2007). Simulation studies have indicated that the VLMR test favors selection of more components when used with small samples, resulting in increased Type I error rates; this suggests the need for an adjusted test (aVLMR) with samples less than 300 (Lo et al., 2001). For our purposes, we chose to rely on the BIC.

Note that our data had an inherent dependency—the nesting of multiple measures through time within each agent. Ignoring a data dependency is known to produce biased standard errors with large alpha inflation as the common result (Cohen et al., 2003). However, current mixture modeling practices that incorporate methods for accounting for the dependency preclude any descriptions of predictors. In this case, that would result in the loss of the means and variances for the position factors that detail key information about the basins of attraction. We therefore chose to temporarily ignore the dependency, recognizing that the standard errors for each coefficient may be biased toward Type 1 errors.

To better understand the extracted equation groups, we saved out the *posterior probabilities* for each data vector. This is the probability that each instance in time for a given agent belonged to one of the classes characterized by a particular equation set where the set of posteriors for a given vector sum to one. It is the equivalent of factor scores if mixture groups as likened to a categorical latent variable. The value of the posterior probabilities is that they allow us to specifically link each agent to the various attractor dynamics at each point in time. Through the combination of the description of each attractor dynamic and the posterior probabilities linking the agents to the topologies, we are able to traverse between the observed vectors from the agents to the underlying topology.

### One dimensional systems

What follows is an illustration of the analytic strategy for the flocking example using the headings from all agents. Fit indices of the 300 flocking agents over 1,000 iterations resulted in sixteen unique attractors (as indicated by the BIC at its lowest value). Table 1 contains the estimated parameters for each of the sixteen equations. All sixteen patterns are attractors as indicated by the negative slopes. They vary in their stability, indicated by the range of slopes. The headings to which each pattern indicates a point of attraction is identified by converting the intercepts and slopes into the set point (−*b*_0_/*b*_1_). In essence, the flock example is characterized by a total of sixteen unique attractors.

**Table 1:** **Unstandardized coefficients from the sixteen attractor solution for the Flocking model of headings**.

**Pattern**	**Intercept**	**Slope**
1	348.528 (1.485)	−1.428 (0.007)
2	300.932 (1.632)	−1.402 (0.007)
3	235.521 (3.214)	−1.306 (0.013)
4	197.095 (3.055)	−1.202 (0.093)
5	255.672 (1.519)	−1.174 (0.061)
6	147.377 (2.554)	−1.103 (0.014)
7	93.272 (2.171)	−1.063 (0.013)
8	24.897 (0.650)	−1.019 (0.003)
9^*^	*271.455 (1.389)*	*−0.993 (0.005)*
10	332.548 (1.056)	−0.972 (0.006)
11^*^	*259.730 (3.296)*	*−0.952 (0.012)*
12	227.678 (6.830)	−0.926 (0.021)
13	334.308 (0.936)	−0.896 (0.052)
14	21.801 (0.811)	−0.881 (0.037)
15	284.039 (5.719)	−0.818 (0.021)
16	245.613 (7.467)	−0.755 (0.022)

**Figure 2:**
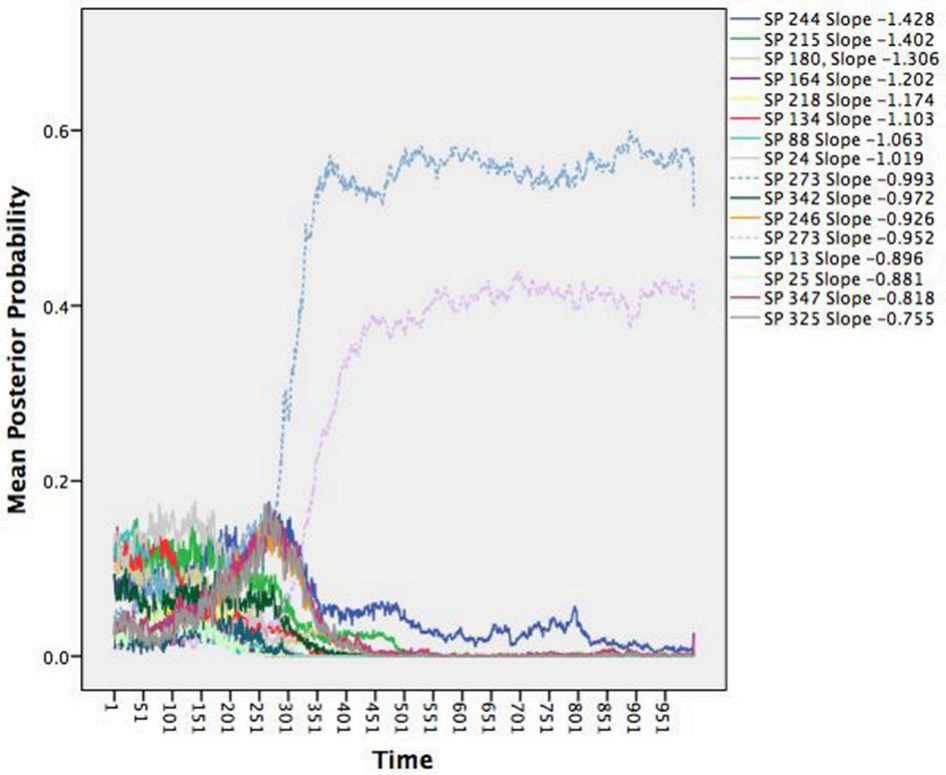
**Time series of the average posterior probabilities for each attractor dynamic pattern**. All patterns were attractors as indicated by their negative slopes, but differed in terms of their set points (SP; the heading in which the agents were attracted toward) and the attractor strengths (the steepness of their slopes). At around iteration 300, the system shows a phase transition wherein two patterns with the same heading begin to dominate.

Patterns ordered by slope deviation from zero (to match Figure 2). Italicized patterns marked with an

* *match dotted patterns in Figure 2*.

We can link the attractors back to the individual agents through the posterior probabilities. For purposes of relating to the initial assessment of the many unique patterns dying off, we chose to illustrate the average posterior probabilities (the average likelihood a given agent is depicted by a given attractor) as a function of time. Figure 2 shows the average posterior probabilities for each attractor dynamic. The legend shows the heading attracted to (set point) and level of attraction (slope) as a function of time. Consistent with Figure 1 (and expectations), initially there were many attractors, but somewhere around iteration 300, two specific attractors started to dominate (dotted lines in Figure 2).

Notice that they share the same heading of 273 degrees, but with slightly different degrees of attraction. Recall the three rules that constitute the changes in heading over time: alignment, separation, and cohesion. Alignment and cohesion drive the agents toward a single heading, but separation instead evokes divergence when agents become too close (and specifically overrides the other two rules). What distinguishes the patterns is not the heading they are drawn toward, but in the divergences themselves due to separation that produces a weaker attractor. Note that agents can be switching between the two attractors over time moving to the slightly weaker attractor, as they need to avoid collisions.

We gain additional information from the quantitative attractor dynamic description as illustrated in Figure 2 when compared to Figure 1. Each data vector is now depicted not only in terms of its vector, but also the likely attractor in which it is drawn (through the posterior probabilities). Further, the description is now in terms of the underlying system forces that depict the type of pattern (all attractors since all the slopes were negative), the location to which the patterns are relative (the set points), and their stability under perturbations (the deviation of the slopes from zero). However, thinking topologically becomes even more beneficial as we move toward systems with more dimensions.

### Two dimensional systems

Modeling a two dimensional system can be captured through two simultaneous equations.

(2) $$\begin{array}{r} {ẋ}_{t}=b_{0}+b_{1}x_{t}+b_{2}y_{t}+e_{xt} \end{array}$$

(3) $$\begin{array}{r} {ẏ}_{t}=b_{3}+b_{4}x_{t}+b_{5}y_{t}+e_{yt} \end{array}$$

These equations represent two variables measured simultaneously (x and y) at time t, x-dot and y-dot are their estimated derivatives at time *t*, *b*_0_ and *b*_3_ are intercepts, *b*_1_ and *b*_5_ are each variable predicting its own derivative, *b*_2_ and *b*_4_ are crossover or coupling relationships, and *e*_*xt*_ and *e*_*yt*_ are errors in equation. Notice that Equation (2) is identical to Equation (1) with the addition of the other changing variable also predicting velocity in x (or x predicting velocity in y). By having both variables changing simultaneously, we generate a two dimensional depiction. The emergent dynamic (attractor, repeller, etc.) is a function of all the *b* coefficients in the equations (Gottman et al., 2002). Common interpretation is that the *own effects* (i.e., x predicting change in x and y predicting change in y) depict the stability properties of the dynamic pattern (attractor, repeller, or saddle) such that negative coefficients are indicative of attractive behavior and positive coefficients are indicative of repulsive behavior in the respective dimensions. The crossover relationships (also known as *coupling effects*) are commonly interpreted to represent the push-pull of variables that constitute cycles and swirling qualities graphically. The set point is a function of both equations. And as noted earlier, two-dimensional systems can include saddles and cycles, which are topological features that are not possible in one-dimensional systems.

While many cases can be interpreted as described in the previous paragraph, some cases do not always conform to the conventional interpretations (and we include some examples of this below). A common violation relates to the notion of collinearity. If all variation in both x and y perfectly map onto one another, then x and y are essentially a single dimension. Under this circumstance the coefficients can be misrepresentative of the dynamic pattern. In our spatial movement circumstance, agents will sometimes capitalize on diagonal movement as a primary, singular dimension. Assessment of the eigenvalues and eigenvectors of the coefficients (treated as a Jacobian matrix of partial derivatives for estimating local Lyapunov exponents; Arabanel et al., 1992) is a method for verifying and determining whether to follow the classic interpretation or whether the interpretation should be modified.

#### The ants model

**Figure 3:**
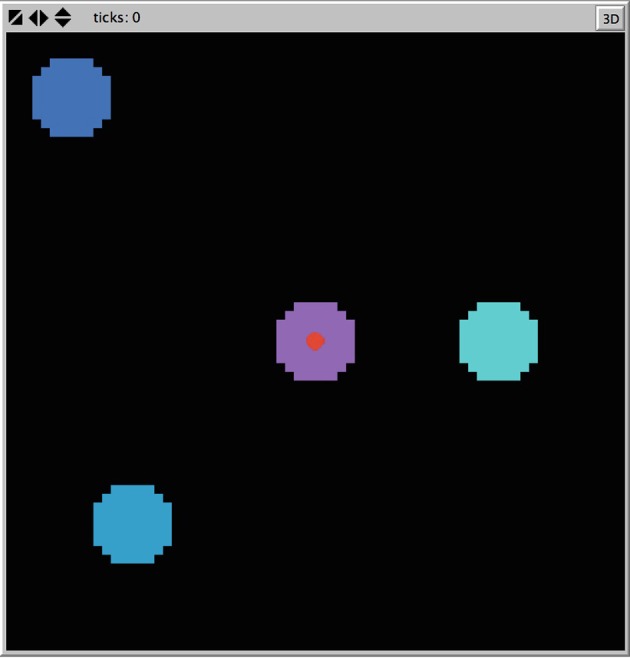
**Screenshot of nest and food placement of the Ants model from Netlogo**.

Consider the Ants model (Wilensky, 1997) in NetLogo v5.2.1 (Wilensky, 1999). This agent-based model was designed to simulate ant colony foraging behavior. The simulation consists of 125 ants each with the same instructions, starting at a nest in the center of a two-dimensional space. Ants are released one at a time from the nest, moving at a constant velocity. Three food sources are placed within the two-dimensional space each with a finite quantity of food supply. The ants search the environment for food (following a random direction algorithm) and upon locating and collecting food, return it to the nest. The primary mechanism for the emergent foraging behavior involves the ants releasing digital pheromones while carrying food and that the ants are attracted to this pheromone. This is much like how stigmergy, a form of environmental modification by individual social animals that affords collective coordination, is proposed to work in live ant populations (Theraulaz and Bonabeau, 1999). The nest also releases a pheromone signal so that the ants can find the nest. The simulation allows for the manipulation of the evaporation and diffusion rates of the pheromones, which we left at default settings. Figure 3 shows the standard placement of food sources in the environment in relation to the nest at the center.

From visual inspection, several emergent colony behaviors can be observed. Ants will search the environment until a critical threshold of ants find a given food source. At this point the ants will form a trail between the food source and the nest. There are sometimes congestion-like behaviors that occur in the middle of the trail or near the nest as more ants converge toward the strongest pheromone locales. Once the food source is used up, the ants once again spread out into a search pattern until a new food source is found. In this case, we will depict the attractor dynamics of the ant movement in two dimensions as a way to characterize the different ant behavioral patterns.

**Figure 4:**
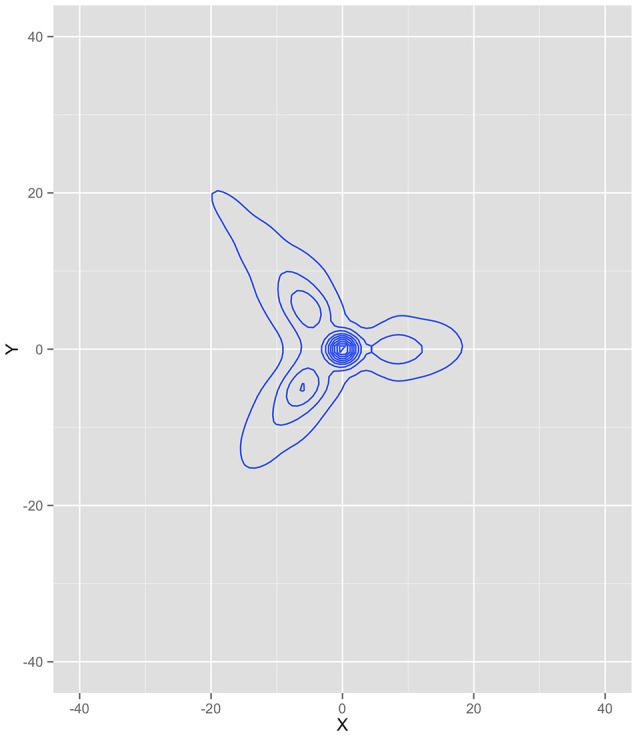
**Kernel density plot of where the ants spent most of their time during the simulation**. Note that the highest densities correspond to the three food source locations and the nest.

We extracted the horizontal (x) and vertical (y) coordinate position of every ant from the beginning of the simulation until the last food pile was fully exhausted, totaling 1,080 iterations. Figure 4 is a kernel density plot of the ant positions, collapsed across all ants and all iterations. This shows the regions where ants spent most of their time and can be thought of as the probability density function of the data (under the assumption of two dimensions)—a graphical illustration of the integral of the dynamics. The density plot is read in the same fashion as a topographical map, where the lines illustrate more density. Note that the greatest density is at the nest (0,0). This was likely a function of all the ants starting at the nest, including the dispersion algorithm of only a single ant leaving the nest per iteration. It is also a function of all the ants returning to the nest to deliver food. Each branch of the density plot corresponds to one of the food sources, consistent with a trail between the nest and the food source. The densest part for each of the branches was, however, closer to the nest than the food source.

**Figure 5:**
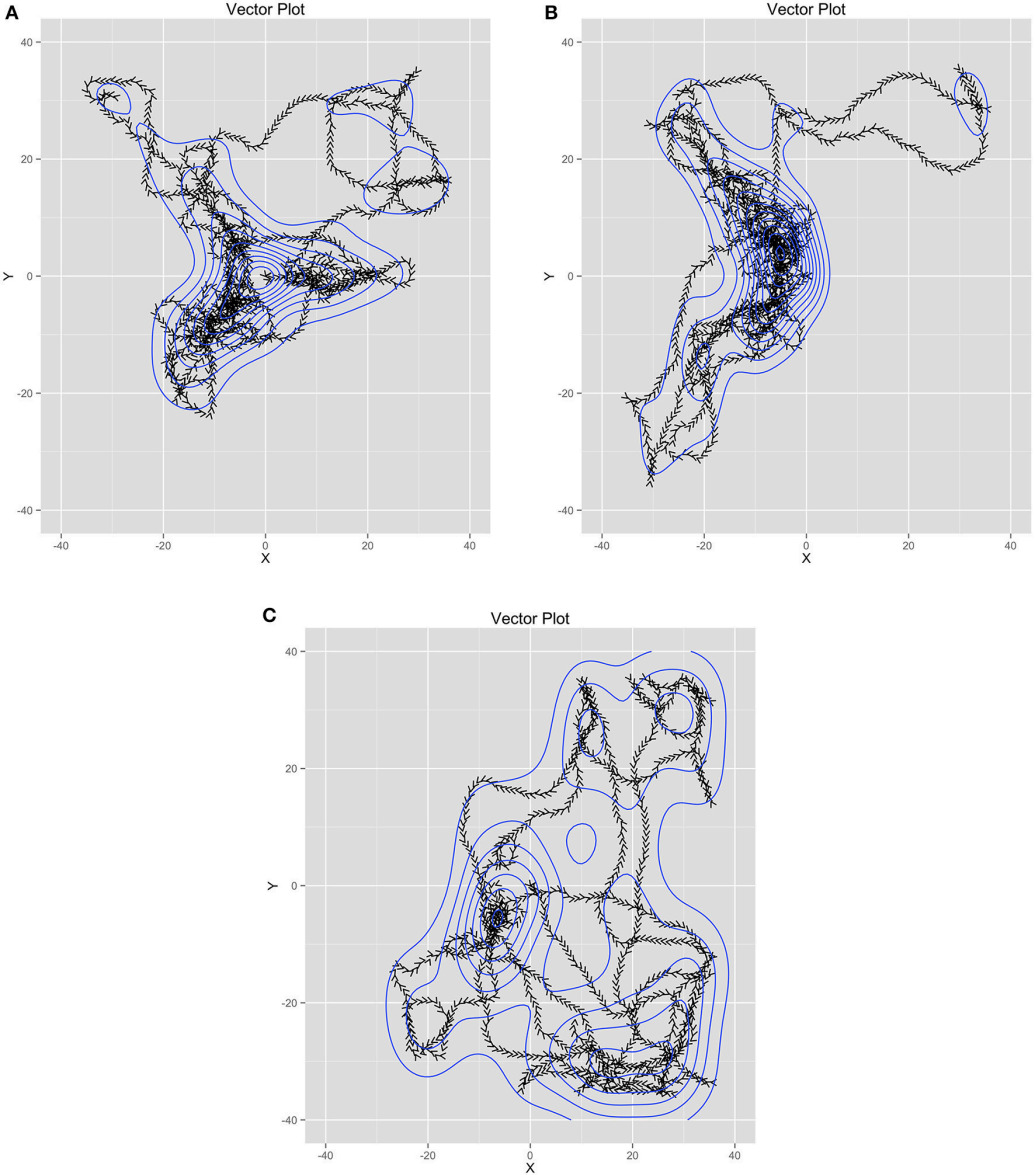
**(A-C)** Three example ant trails that illustrate how the ant behavior is shared across all the ants while each ant had unique behavior.

Figure 5 contains trails of three exemplar ants as vector plots in time to help illustrate the link between individual agents and the model estimated from all agents. Figure 5A shows the trail of an ant that helped collect food from all of the food piles. However, it also shows searching behavior in some of the areas of the world where food did not reside. Figure 5B illustrates an ant that only helped collect food from two piles and also participated in searching behavior in empty quadrants of the world. Figure 5C shows an ant that participated minimally in food collection instead spending more time searching. As a whole, these illustrate that the emergent behavior is not from any one ant. Instead, it is through their interactions with one another (through pheromones) and the environment (food resources relative to the nest) that their behavior becomes emergently coordinated.

**Figure 6:**
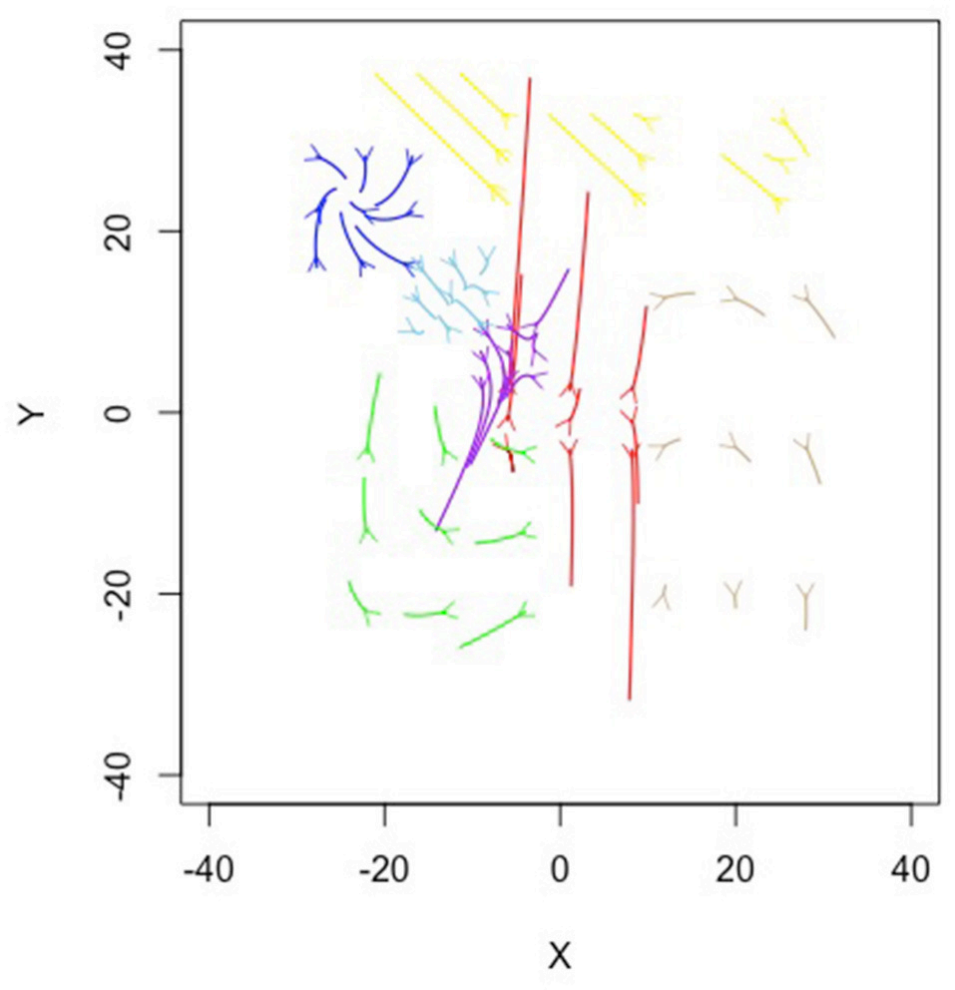
**Topographical illustration of the seven equation solution for the Ant simulation**.

Our mixture model identified a total of 7 different patterns in the example ant model (minimized BIC at 7 groups). Table 2 shows all the coefficients for the seven different patterns, labeled by their colors from Figure 6. The last two columns are the eigenvalues wherein we built matrices of the own and coupling effects in the same order as Equations (2) and (3) (First row: own predicting x, coup predicting x and second row: coup predicting y, own predicting y). The eigenvalue procedure allows us to account for when the coefficients do not directly represent the type of attractor dynamic due to the primary axes for the dynamic depictions being different from the variables used in the equations. When the eigenvalues are both real numbers and negative, the system depicts an attractor. When the eigenvalues are both real and positive, the system depicts a repeller. When one is positive and one is negative, the system depicts a saddle. Imaginary numbers instead depict cyclic behavior with complex numbers being a combination of cyclic and attractive/repulsive at the same time (Abraham and Shaw, 1983).

**Table 2:** **Unstandardized coefficients (and standard errors) for the seven group solution along with eigenvalues for the Ants model**.

**Pattern**		**Own**	**Coupling**	**Intercept**	**Eigenvalues**
Blue	X	−0.009 (0.002)	0.006 (0.002)	−0.361 (0.064)	−0.011 + 0.007i
	Y	−0.013 (0.001)	−0.009 (0.002)	0.096 (0.064)	−0.011 − 0.007i
Light Blue	X	−0.020 (0.003)	0.018 (0.004)	−0.467 (0.075)	−0.040
	Y	−0.023 (0.004)	0.020 (0.004)	0.522 (0.083)	−0.002
Purple	X	−0.014 (0.003)	0.014 (0.004)	−0.146 (0.035)	−0.028
	Y	−0.001 (0.005)	0.027 (0.004)	0.107 (0.049)	0.013
Yellow	X	0.005 (0.001)	0.018 (0.003)	−0.645 (0.071)	−0.011
	Y	−0.017 (0.003)	−0.005 (0.001)	0.605 (0.085)	−0.000
Green	X	−0.003 (0.001)	0.002 (0.001)	−0.049 (0.014)	−0.001
	Y	0.003 (0.001)	−0.004 (0.001)	0.002 (0.014)	0.001
Brown	X	0.000 (0.001)	0.001 (0.000)	0.022 (0.019)	0+0.002i
	Y	0.000 (0.000)	−0.003 (0.001)	0.046 (0.017)	0−0.002i
Red	X	0.000 (0.001)	0.001 (0.002)	0.011 (0.004)	0.019
	Y	0.019 (0.002)	−0.006 (0.001)	0.036 (0.004)	0.000

Figure 6 is a topographical representation of the seven attractor dynamics patterns emergent in the ant behavior. Figure 6 was generated by using the estimated equations from the mixture model in conjunction with the adaptive Runge-Kutta algorithm from the deSolve package (Soetaert et al., 2010) in R (R Core Team, 2016) to estimate example trajectories iterated over time. In each case, values were chosen using the position means and variances extrapolating in all possible combinations of one standard deviation in X and Y and iterating the trajectories forward in time. Details on each pattern follow.

The blue, brown, and green patterns correspond to the food piles while the red pattern corresponds to the ant nest. The yellow pattern corresponds to searching an area where no food existed. The light blue captured the pattern of the ants converging in the middle of the trail as the pheromones were most intense there and the purple captured the dispersal after the food pile in the upper left had been fully collected (it was the first pile found in the simulation).

Notice how each pattern is captured through a different attractor dynamic. For example, the red nest pattern shows a repeller in which ants leave the location. If we capture each ant trail of food collection through the other patterns, then what primarily remains is the initial leaving from the nest. The blue and brown patterns, both corresponding to food piles, show cyclic properties (they have imaginary components to their eigenvalues). This is capturing the pattern of getting the food from the pile, bringing it to the nest and returning. The pattern corresponding to the lower left food pile was a saddle, however—attractive in one dimension and repulsive in the other. By having the set point far from the dynamic pattern, the saddle generated curved trails that could then be completed by feeding into other, already established, patterns.

**Figure 7:**
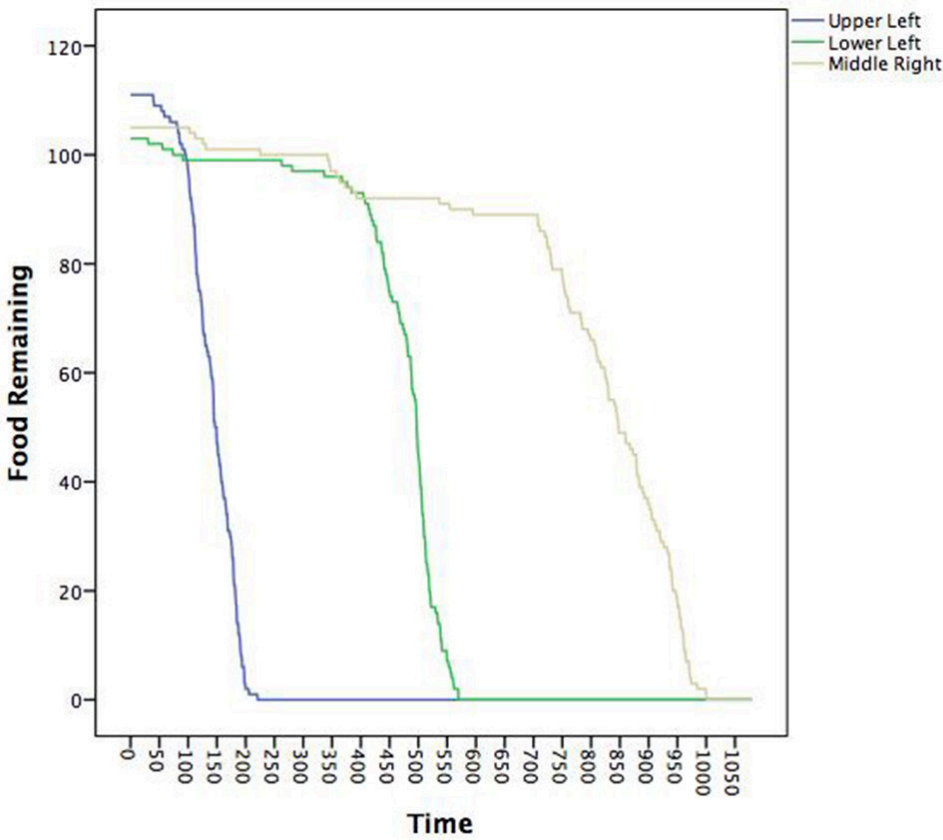
**Time series plot of the amount of food available in each of the three food piles**. The legend describes where in the coordinate space a given food pile was located (see also Figure 3).

Now, we link the agents to these patterns and to key system descriptions—in this case food depletion. Figure 7 shows the decline in the food piles as a function of time. Notably, the ants found the pile in the upper left first, followed by the lower left and then finally the middle right. We ran seven multilevel models treating the posterior probabilities of each pattern as the outcome as a function of the proportion of food remaining in each pile (a three predictor MLM). The fixed and random effects along with intraclass correlations (ICC) are in Table 3. All random effects were significantly non-zero suggesting that there was variability in their likely pattern as a function of the remaining food piles among the individual ants. The fixed effects can be interpreted as whether or not the likelihood of being in a pattern occurred where a positive sign meant that declines in a food pile corresponded to declines in the pattern and a negative sign meaning that declines in a food pile corresponded to increases in the pattern. Given the order of the food pile depletions, the pattern of effects can also roughly determine when the pattern was more prevalent.

**Table 3:** **Unstandardized coefficients (and standard errors) and intraclass correlations from multilevel models predicting the posterior probabilities of being in each of the seven groups for the Ants models**.

	**Upper left food pile**	**Lower left food pile**	**Middle right food pile**	**Intercept**	**ICC**
**FIXED EFFECT**					
Blue	0.014 (0.009)	−0.018 (0.010)	−0.248 (0.020)^*^	0.269 (0.016)^*^	0.046
Light Blue	−0.008 (0.006)	0.008 (0.008)	−0.239 (0.013)^*^	0.249 (0.011)^*^	0.031
Purple	−0.019 (0.005)^*^	−0.010 (0.009)	−0.221 (0.015)^*^	0.259 (0.012)^*^	0.021
Yellow	−0.020 (0.011)	−0.018 (0.010)	0.034 (0.011)^*^	0.028 (0.007)^*^	0.185
Green	−0.092 (0.024)^*^	−0.304 (0.023)^*^	0.479 (0.029)^*^	0.025 (0.017)^*^	0.070
Tan	−0.219 (0.025)^*^	0.187 (0.025)^*^	0.072 (0.027)^*^	0.094 (0.018)^*^	0.116
Red	0.345 (0.025)^*^	0.154 (0.011)^*^	0.123 (0.012)^*^	0.076 (0.006)^*^	0.020
**VARIANCE COMPONENT**					
Blue	0.009 (0.001)^*^	0.012 (0.002)^*^	0.049 (0.006)^*^	0.033 (0.004)^*^	
Light Blue	0.004 (0.0010^*^	0.008 (0.001)^*^	0.021 (0.003^*^	0.014 (0.002)^*^	
Purple	0.003 (0.000)^*^	0.009 (0.001)^*^	0.029 (0.004)^*^	0.017 (0.002)^*^	
Yellow	0.014 (0.002)^*^	0.012 (0.001)^*^	0.016 (0.002)^*^	0.006 (0.001)^*^	
Green	0.072 (0.009)^*^	0.063 (0.008)^*^	0.107 (0.014)^*^	0.037 (0.005)^*^	
Tan	0.080 (0.010)^*^	0.079 (0.010)^*^	0.087 (0.011)^*^	0.039 (0.004)^*^	
Red	0.079 (0.010)^*^	0.014 (0.002)^*^	0.017 (0.002)^*^	0.004 (0.001)^*^	

* *Denotes p < 0.05*.

The red pattern at the nest was likely when all the food sources were untouched and declined in likelihood as all the food piles declined, consistent with the ants initially leaving the nest to search. The blue, light blue, and purple patterns all associated with the upper left quadrant were all less likely when the last food pile was untouched, but only the purple (the theoretical dispersion after the food pile was depleted) was contingent upon the corresponding upper left food pile. The negative sign was indicative that declines in the first pile increased the likelihood of the purple dispersion pattern consistent with leaving the trail to find another food source once the food in the first pile was depleted. The green (corresponding to the lower left food pile) and brown (corresponding to the middle right food pile) patterns were predicted by all three food piles with negative coefficients suggesting that as any food depleted, these became more likely—consistent with these food piles being found later. Finally, the yellow pattern was only uniquely predicted by the middle right food pile depletion such that as the food pile declined, so did the likelihood of being in the search pattern. Given that as more ants found the last food pile, more converged on it. Once it depletes, however, fewer ants would be in this search pattern.

#### Baboons navigation data

**Figure 8:**
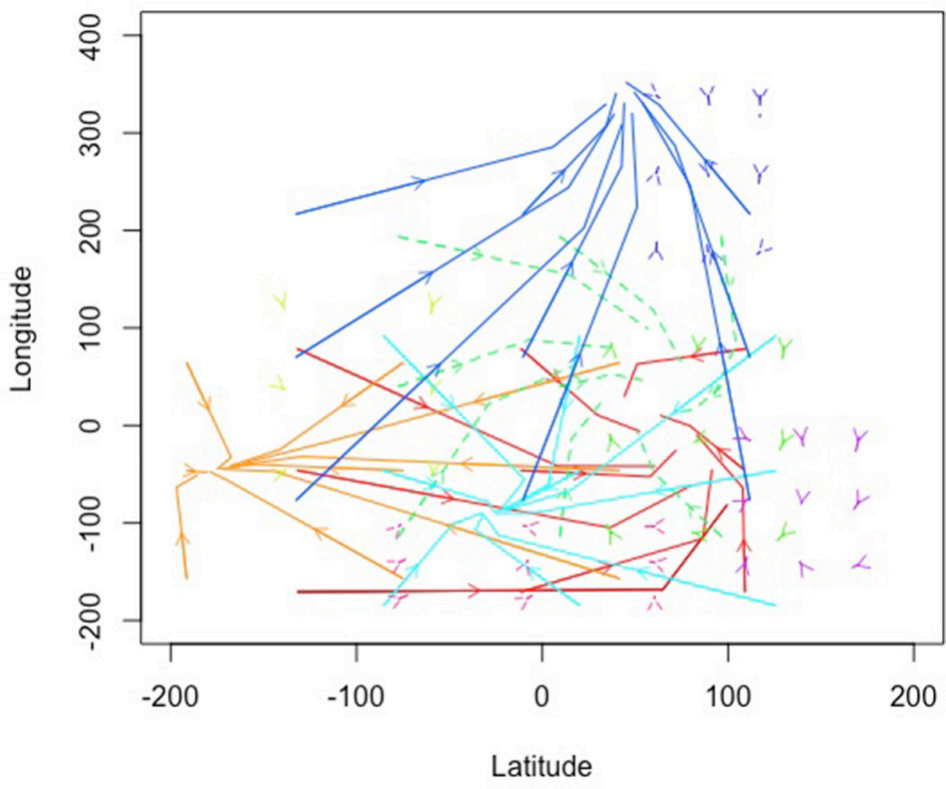
**Topographical solution for the Baboon gps data**.

So far, we have relied on simulations to illustrate how one can depict higher order emergent coordination for agent interactions using attractor dynamics. Our next two examples are derived from observed data. Figure 8 represents a solution from global positioning system (GPS) data collected from a troop of baboons at the De Hoop Nature Reserve in South Africa. Table 4 contains the coefficients and eigenvalues, again using colors to indicate correspondence. To collect this data, researchers recorded the positions of 14 adult baboons by holding a GPS device over or very close to each animal at different points over a 74 day period (data was made available by Bonnell et al., 2016; and further details of the original study can be found at Bonnell et al., 2017). Consistent, with the ants data, this example data is in an x/y coordinate space, but now in longitude and latitude. To facilitate estimation due to variability occurring in small decimal places, longitude and latitude were mean-centered and multiplied by 1,000.

**Table 4:** **Unstandardized coefficients (standard errors) and eigenvalues for the 10 pattern solution from the Baboon GPS data**.

**Pattern**		**Own**	**Coupling**	**Eigenvalues**
Red	X	−0.821 (0.015)	−0.280 (0.013)	−0.554 + 0.441i
	Y	−0.314 (0.023)	0.689 (0.023)	−0.554 − 0.441i
Orange	X	−0.687 (0.021)	0.084 (0.017)	−1.713
	Y	−0.860 (−0.14)	−0.140 (0.039)	−0.828
Yellow	X	−0.025 (0.002)	−0.007 (0.001)	−0.178
	Y	−0.005 (0.001)	−0.019 (0.002)	−0.004
Light Green	X	−3.720 (0.637)	−0.906 (0.182)	−0.006 + 0.017i
	Y	1.595 (0.455)	3.798 (1.660)	−0.006 − 0.017i
Dark Green	X	−0.024 (0.003)	0.005 (0.001)	−0.424 + 0.311i
	Y	−0.011 (0.001)	−0.062 (0.003)	−0.424 − 0.311i
Light Blue	X	−0.386 (0.015)	0.164 (0.015)	−1.205 + 0.350i
	Y	−1.073 (0.036)	−1.103 (0.032)	−1.205 − 0.350i
Blue	X	−1.066 (0.019)	−0.039 (0.017)	−0.857 + 0.239i
	Y	−1.040 (0.040)	0.807 (0.047)	−0.857 − 0.239i
Navy Blue	X	−0.011 (0.005)	0.002 (0.002)	−0.026 + 0.011i
	Y	−0.035 (0.003)	−0.109 (0.009)	−0.026 − 0.011i
Purple	X	−0.008 (0.030)	0.014 (0.014)	−0.028 + 0.017i
	Y	−0.035 (0.019)	−0.064 (0.039)	−0.028 − 0.017i
Magenta	X	−0.013 (0.002)	−0.020 (0.003)	−0.026
	Y	−0.014 (0.003)	−0.007 (0.002)	−0.002

Figure 8 illustrates that several of the patterns show cyclic behaviors. In fact, all the eigenvalues were negative with 7 of the 10 showing imaginary eigenvalues consistent with cyclic behaviors. Further, all patterns had at least one negative real eigenvalue suggesting that they all were attractive indicating a pattern of convergence for baboons. Figure 8 clearly shows that the patterns were not equally attractive, however, in that vector length differed dramatically when example trajectories were estimated. This can also be seen by the size of the eigenvalues where some were quite close to zero in their real number portion(s) while others were much smaller numbers approaching and surpassing negative one. Thus, some of these patterns were more stable clusters for the baboons while others were more loose associations around the shared longitude/latitude set point.

**Figure 9:**
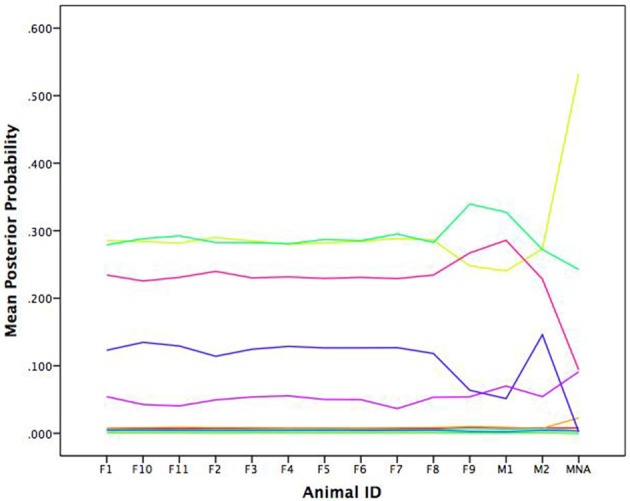
**Average posterior probabilities associated with each equation group, by baboon**. F is for female and M is for male.

In their original work, Bonnell et al. (2017) evaluated whether the movement patterns of a focal individual baboon was influenced by the location of the troop as a collective or by the locations of specific influential members of the troop. Ultimately, their results showed evidence for both of these patterns. In some cases, the focal baboon's movement was highly influenced by the average movement location of the entire troop. In other cases, the focal baboon's movement was quite sensitive to the movements of the alpha female (F1) and the alpha male (M1). To link back to individual baboons, our results suggest a consistent pattern as illustrated in Figure 9 wherein we show the average posterior probabilities for each baboon illustrating which pattern would arguably influence a given baboon the majority of the time (again, colors correspond). Few distinctions existed between the female and dominant male baboons showing preference for the light green (cyclic attractor) and yellow (attractor) patterns. The dominant male (M1) showed slightly more preference for the magenta pattern (also an attractor). Thus, there is evidence of following the primary male baboon, but also one of a female majority. And yet in both cases these most common patterns represent the least attractive patterns (eigenvalues closest to zero) in that there is lots of wandering in comparison to the other patterns inferred from the GPS data.

### Beyond two dimensions

As we move beyond two dimensions, it is difficult to make easy to read and meaningful maps of the data. However, our approach is not limited to two dimensions. By relying on the eigenvalues presented earlier, one can derive the higher order patterns to illustrate what is occurring without a means to draw them. Further, it also allows us to point out that any variables can be captured as attractor dynamics—they do not inherently need to be spatial, as illustrated by our next example.

Each new dimension corresponds to an additional equation. In the six-dimensional case that follows, we model six simultaneous equations where change in each variable is treated as the outcome from each equation. Further each variable at a given point in time is allowed to freely predict the changes in each equation. The matrix used to generate the eigenvalues is based on the coefficients where, as before, the main diagonal are the own effects and the off diagonals are the coupling relationships. Each matrix row corresponds to a different equation.

#### Affect in families data

To show a non-spatial example with more than 2 simultaneous change equations, we modeled positive and negative affect from the PANAS (Watson et al., 1988) taken from mothers, fathers, and adolescents from 252 families where the adolescent has type 1 diabetes. The data are taken from the Adolescents with Diabetes and Parents Together study where each family member completed a daily diary for 14 days (further study details can be found at Berg et al., 2009).

We extracted two stable patterns (three patterns would not properly converge and fit indices supported the two pattern solution). Table 5 provides the estimated coefficients. Notably, the eigenvalues were quite different between the two patterns. The first pattern generates all negative eigenvalues indicating that it forms one large six dimensional attractor (−0.709, −0.522, −0.455, −0.428, −0.285, −0.257). The second pattern, on the other hand had complex numbers for the first two eigenvalues suggesting cyclic behavior as a primary component (−0.613+0.027i, −0.613 −0.027i, −0.420, −0.343, −0.180, −0.157).

**Table 5:** **Unstandardized coefficients (and standard errors) from the two pattern solution for the Affect Daily Diary**.

		**Mother +**	**Mother −**	**Father +**	**Father −**	**Adolescent +**	**Adolescent −**
1	ΔM+	−0.39 (0.03)	0.09 (0.03)	0.07 (0.03)	0.08 (0.04)	0.01 (0.02)	0.02 (0.02)
	ΔM−	0.06 (0.02)	−0.55 (0.03)	0.023 (0.03)	−0.11 (0.04)	0.04 (0.02)	−0.04 (0.02)
	ΔF+	0.09 (0.03)	0.05 (0.03)	−0.41 (0.03)	0.09 (0.04)	−0.01 (0.02)	0.02 (0.03)
	ΔF−	−0.00 (0.02)	−0.05 (0.02)	0.08 (0.02)	−0.55 (0.04)	0.02 (0.02)	−0.03 (0.02)
	ΔA+	0.01 (0.04)	0.09 (0.04)	0.02 (0.04)	0.09 (0.06)	−0.41 (0.03)	0.12 (0.03)
	ΔA−	0.00 (0.03)	−0.09 (0.03)	0.01 (0.03)	−0.08 (0.02)	0.09 (0.02)	−0.34 (0.03)
2	ΔM+	−0.33 (0.03)	0.28 (0.12)	0.07 (0.03)	−0.19 (0.21)	0.02 (0.02)	0.03 (0.08)
	ΔM−	−0.00 (0.01)	−0.61 (0.04)	−0.02 (0.01)	−0.04 (0.07)	0.01 (0.01)	0.04 (0.02)
	ΔF+	0.04 (0.03)	−0.03 (0.09)	−0.19 (0.04)	−0.32 (.19)	0.02 (0.02)	0.02 (0.07)
	ΔF−	−0.01 (0.01)	0.01 (0.02)	0.00 (0.01)	−0.50 (0.07)	0.00 (0.00)	−0.01 (0.01)
	ΔA+	0.06 (0.03)	0.36 (.12)	0.01 (0.03)	0.35 (.24)	−0.20 (0.02)	0.19 (0.09)
	ΔA−	0.00 (0.01)	0.00 (0.05)	−0.01 (0.01)	−0.12 (0.08)	0.02 (0.01)	−0.41 (0.06)

*In the form of matrices used to estimate eigenvalues. Table was rounded to second decimal for space. Rows are changes (Δ) in Mother (M), Father (F), and Adolescent (A)*.

**Figure 10:**
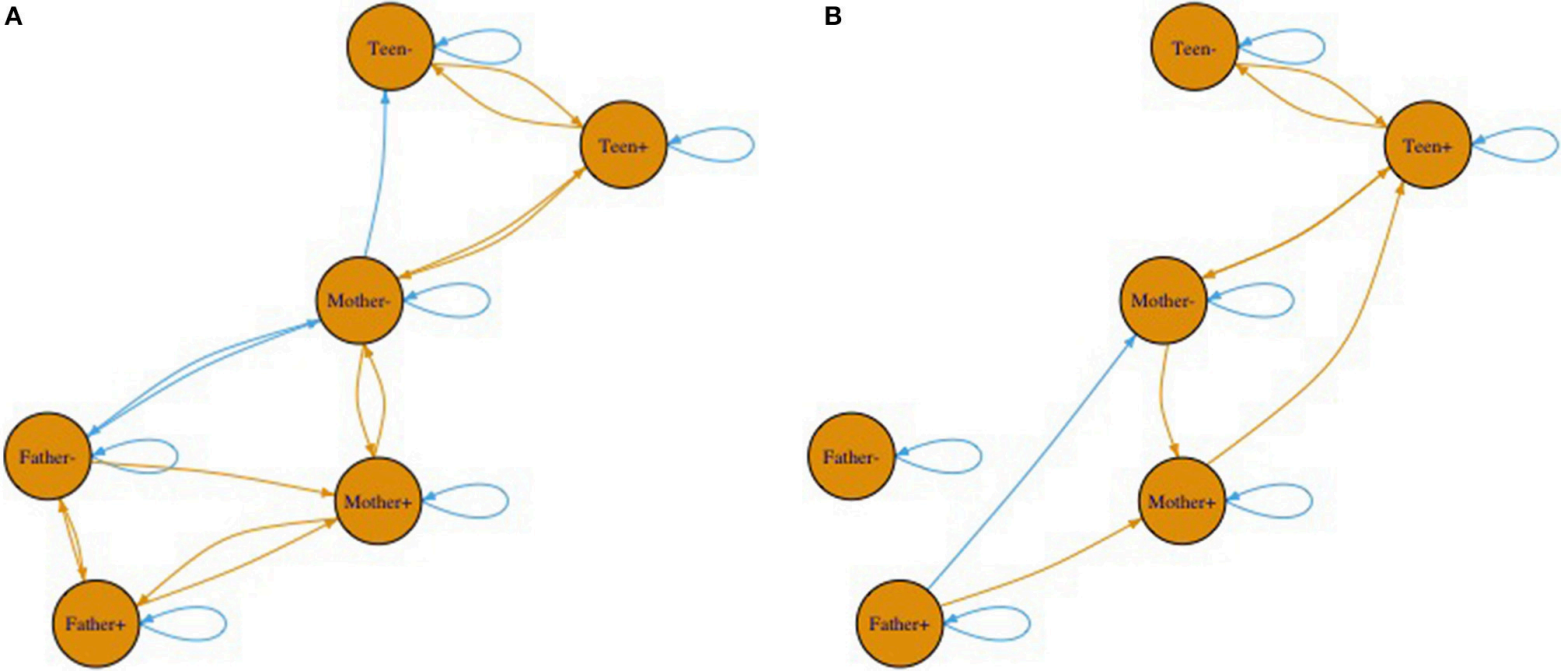
**(A,B)** Two network diagrams that illustrate the two different equations. Beginning of arrows represent value at time t. Arrow heads represent change in value.

Though we cannot draw a map to represent this higher order pattern, one way to represent the changes in the system is through a network diagram. Figures 10A,B shows only significant (alpha = 0.05, two-tailed) pathways between affect variables. The beginning of an arrow is value and the end of an arrow is change. Blue arrows represent negative relationships and brown ones are positive. Note between Figures 10A,B the connections between individuals breaks down substantially with the cyclic nature relating to the less connected network. The most noteworthy is the changing connections of father's affect to the mother and adolescent. It is noteworthy that these coefficients merely indicate prediction and thus any interpretation of causality would overstate the relationship. That said, fathers were clearly showing less connection in the second pattern.

To link back to individual families, we built a multilevel model predicting the posterior probabilities for the first pattern as a function of diabetes risk for the adolescent on a given day. We use the variable *risk* as an easy to interpret indicator as to how well the adolescent was managing their diabetes on a given day. Risk is a rescaled version of daily blood glucose variability and level such that zero indicates perfect maintenance at doctor recommended levels and 100 indicates either going too high or too low repeatedly (both of which can be quite dangerous; see Kovatchev et al., 2006). Since posterior probabilities for a given data vector add to one, high probabilities of being in the first pattern inherently implies a low probability of being in the second. Table 6 contains the coefficients. At zero risk on a given day, families were equally likely to be in each pattern (the intercept is the posterior probability when risk was zero). As risk increased, however, families were more likely to fall into the second pattern. That is, on good days we see the more connected attractor pattern and on bad days the father appears less connected and the family affect adopts a cyclic attraction pattern instead.

**Table 6:** **Unstandardized coefficients (and standard errors) from multilevel model predicting the posterior probability of the first pattern as a function of Diabetes risk**.

	**Fixed coefficients**	**Variance components**
Intercept	0.502 (0.027)^*^	0.100 (0.012)^*^
Risk	−0.003 (0.001)^*^	0.000 (0.000)

* *Denotes p < 0.05*.

## Discussion

Kelso (2009) posited that SCD “unites the spontaneous, self-organizing nature of coordination and the obviously directed, agent-like properties characteristic of animate nature into a single framework” (p. 1540). This logic matches with self-organization from agent-based models, and cases where many agents engage in social coordination, more generally. By connecting attractor dynamics modeling with cases where there are a range of agents and a range of outcomes allows for a generalized approach to quantifying the emergent patterns.

Through various examples, we illustrated that the attractor dynamics can be captured using a combination of difference/differential equation modeling and mixture modeling. Further, we showed that these attractor patterns and their occurrence could be linked with different outcomes. For the flocking model, we found sixteen attractor patterns of the agents' heading that converged on fewer attractors over time. For the ants model, we found seven dynamic patterns to depict their motion in a two-dimensional x/y space that roughly corresponded to qualitative depictions of rules the ants follow. For the baboon navigation data, we found ten patterns in two-dimensional longitudinal and latitudinal space in which the probability of exhibiting a particular attractor was contingent upon influential baboons in the troop (e.g., an alpha male). For families where an adolescent has type 1 diabetes, we found two patterns in a six dimensional affect space that corresponded to higher and lower levels of risk from the disease. By using the data from all the agents, the underlying topology is inclusive of all the agents. In the ants model, for example, not all ants illustrated being influenced by every pattern. Instead, ants can exist in a single pattern their entire time or move between them. Thus, the underlying map implied by the set of dynamic patterns generates an inclusive generalization both within and between agents that capitalizes on the most probable systems states over the duration of the observation period.

In each circumstance, the technique depicts the topological feature in terms of the implied patterns and the stability of those patterns. Whereas, the flocking model only contained attractors that varied in their set points (attractive headings) and their stabilities, the ants model illustrated all the common possible attractor dynamic patterns including attractors, repellers, saddles, and cycles.

The complexity of the underlying pattern is directly related to the number of dimensions. With a single dimension, attractor dynamics may only convey attractors and/or repellers. With two dimensions, cycles and saddles can be inferred. Beyond two dimensions, chaotic (strange) attractors are possible, though all currently known chaotic attractors require non-linear equation forms and the equations herein were restricted to linearity within each equation group. Thus, this is a limitation of the technique provided.

In each case, we then linked the quantification back to the individual agents. Through mixture modeling we did this by outputting the posterior probabilities. These probabilities are the probability that a given data vector is under the influence of a given dynamic pattern, the probabilities for a given vector sum to one across all the possible patterns. Therefore, these probabilities maintain the data dependency we inherently ignored in the estimation for the dynamic patterns themselves. We therefore always either examined the probabilities at a collapsed agent level (e.g., averages) or through multilevel modeling wherein the dependencies could be properly taken into account. In each case, it could be linked to possible variables of interest used to depict the system. For the headings, this was illustrated with time in that attractors should collapse as time goes on. For the ants model, this was illustrated through food supply. For the baboons, this was illustrated through the location of the alpha male and the females. For affect in families where the adolescent had type 1 diabetes, it was illustrated with the diabetes risk exhibited that day. In all, this allows one to link the higher order patterns back to meaningful outcomes that characterize when agents behave in certain ways or exhibit theoretically important states.

In the spatial examples, we utilized variables that depicted the spatial movement. As an initial foray into understanding attractor dynamics, thinking spatially helps make the concepts more intuitive. But, ultimately, these concepts can be applied in many contexts where relationships are not inherently spatial. Being able to think about the spatial analogs helps ground what is being observed, but does not inherently limit the domains in which attractor dynamics can be examined.

Further, individual equation parameters do not always align with the system depiction graphically or through the eigenvalue procedure. In the ants example, this had to do with the reliance on diagonal movement of the ants. By depicting the system through an equation of x and an equation of y, we mask diagonal movement—it is really a straightforward combination of the two dimensions rather than showing some independence. More generally, the coefficients are under an assumption that the dimensions chosen are the primary dimensions for depicting the changes occurring in the system. The eigenvalue procedure bypasses this assumption by instead capitalizing on dimensions that maximize the strength of the attractor dynamics.

Once we moved beyond two dimensions, the eigenvalue procedure becomes even more valuable. There is no easy way to graphically “see” the implied dynamic, but the sign and distinctions between real and imaginary portions elucidate the attractor pattern. In practice, anytime we model a system with two or more equations we should adopt the eigenvalue procedure as a means to understand the higher order pattern in addition to any interpretations applied to the individual coefficients themselves. For example, it is common to interpret coupling coefficients as the push/pull of one variable upon another. However, this fails to capture what pattern the push-pull creates as their interpretation is under an assumption that we somehow picked ideal dimensions to represent them. Locally, the coefficients maintain their meaning, but we cannot extrapolate the more global pattern of which they are a part.

In regards to equation identification, the technique is not without its limitations. The choice of slicing up the data into a series of locally linear equations is an imperfect method for capturing non-linear dynamic models. Specifically, non-linear dynamic models can have both multistability in which more than one pattern is stable simultaneously and cases where variables differentiate when one pattern is or is not accessible. By slicing up the data into a series of locally linear equations through mixture modeling, these two circumstances are difficult to distinguish. One can begin to distinguish these circumstances by attempting to predict the posterior probabilities. However, ultimately multistability is distinguished by states being probable despite nothing differentiating them (or when the dimensions being examined are all that differentiate them). That is, multistability would occur under a lack of being able to predict differences of when agents would be in one or the other. Thus, this approach provides a limited potential for knowing when multistability exists as opposed to having some variable differentiate them. We may never examine the “right” variable or are instead in the situation of arguing a null finding to support the multistable case.

In contrast, it is possible through a cusp catastrophe model in conjunction with multilevel modeling, for example, to allow for differentiating variables (also known as control parameters) without their identification (Butner et al., 2014b), though knowing which scenario you are observing requires examination of many more qualities than discussed herein (Gilmore, 1981). Further, manifolds (the surfaces implied by topological equations) are smooth, while the mixture modeling approach is more patchwork. We do not know the reach of a given attractor dynamic—we chose to represent each dynamic through one standard deviation in each direction from the means when we utilized the Runge-Kutta algorithm to graph plausible trajectories. Notably the means and standard deviations are specific to each dynamic pattern (allowing some to be large and others to be smaller). However, the boundaries of one pattern to another are truly unknown, requiring some inference.

Notably, SCD has tended to rely on cyclical descriptions to model the rhythmic coordination of social agents. While the modeling approach illustrated herein allows for cycles, it does not assume their existence. The direct equation link is that SCD generally functions with second order equations where the second derivatives (acceleration or change in velocity) are treated as the outcomes. Within our structural equation model, it would be analogous to building a quadratic growth model on Toeplitz data where the quadratic growth latent variable would be the second derivative predicted by the other two latent variables (Butner and Story, 2010). Moving to a second order model automatically implies two dimensions and thus generates cycles. However, it is not without a cost. Specifically, second order modeling in this form assumes that the set point of the cycles must equal zero. Overcoming this assumption is currently something under consideration for modeling dynamic patterns and once resolved will unite these approaches more generally.

## Conclusion

Understanding how large-scale, multi-agent social systems coordinate is challenging and complex. In part, the challenge is due to the fact that there are so many agents, system components, and potential system states that can become coordinated; all of which may change over time (Van Orden et al., 2003). These many components interact generating higher order system behavior that is emergent and dynamic. However, knowing the “Dynamics demystifies…emergence” and it can also provide “basic laws for a quantitative description of phenomena that are observed” (Kelso, 2009; p. 1540). As such, we have expanded on work in SCD by demonstrating the utility of modeling the attractor dynamics of several systems to characterize their higher-order behavioral patterns and showed how these patterns varied over time and could be linked to meaningful aspects of the systems.

Within domains, such as agent based modeling, qualitative depictions of higher-order patterns are often known, but not quantitatively modeled. In SCD, phenomena can be non-rhythmic, and yet dynamically coordinated. They can exhibit stability and multistability. Thus, using attractor dynamic descriptions along with statistical innovations, such as mixture modeling, provide a reasonable solution to understanding the large-scale, multi-agent social coordination. Characterizing the higher order properties of the system in this way forms a foundation for examining the emergent patterns through time in either a confirmatory or exploratory manner. This same technique, as we have shown, can be utilized with simulated as well as observational data.

It is our aim that we recognize that we study systems that are inherently open systems (even though simulations are often closed). By examining part of the system (the variables we measure), unobserved aspects of the system function as perturbations to the system. Thus, a system depicting families is open because we are only examining some of the variables involved. To understand how agents exhibit emergent self-organization and coordination, we have advanced a general quantification that can be applied to a range of social systems, such as two individuals that form a couple up to a crowd's behavior. We hope that the widely applicable techniques will be adopted to advance scientific understanding of SCD.

## Ethics statement

The Adolescents with Diabetes and Parents Together project was conducted in accordance with the recommendations of the University of Utah Institutional Review Board. All subjects gave written informed consent in accordance with the Declaration of Helsinki. The protocol was approved by the University of Utah Institutional Review Board.

## Author contributions

All authors listed, have made substantial, direct and intellectual contribution to the work, and approved it for publication.

### Conflict of interest statement

The authors declare that the research was conducted in the absence of any commercial or financial relationships that could be construed as a potential conflict of interest.
