# Supplementary material for: Modeling Multi-Agent Self-Organization through the Lens of Higher Order Attractor Dynamics
Source: Front Psychol. 2017 Mar 20;8:380. doi: 10.3389/fpsyg.2017.00380 (PMC5357650; doi:10.3389/fpsyg.2017.00380)
Supplement: Supplementary file 1 [file DataSheet1.docx]

Appendix A

TITLE: Syntax for Mixture Modeling in Mplus from Two Simultaneous Equations Example with Two Classes

DATA:

file = "ants.dat";

VARIABLE:

names = ant time x_1 x_2 y_1 y_2;

usevariables = x_1 x_2 y_1 y_2;

missing=.;

class=C(2); !Number of classes

auxiliary=ant time; !Variables not to be included in model, but in any output

ANALYSIS:

type is mixture ; !Call for mixture model

starts = 1000 100; !Random start values

processors = 4 2; !# of Computer processors

!optseed=354624; !one class optimal seed number stored for easy recall;

MODEL:

%Overall%

!Estimate position and velocity for each;

xpos xvel | x_1@0 x_2@1;

ypos yvel | y_1@0 y_2@1;

!With only two timepoints, errors in observed x and y fixed to zero;

x_1@0 x_2@0 y_1@0 y_2@0;

!Intercepts fixed to zero as with growth models;

[x_1@0 x_2@0 y_1@0 y_2@0];

!Relationships;

xvel yvel on xpos ypos;

!Intercepts and means;

[xvel yvel xpos ypos];

!Variances and residual variances;

xvel yvel xpos ypos;

%C#1%

!Include what is free across classes

!Relationships;

xvel yvel on xpos ypos;

!Intercepts and means;

[xvel yvel xpos ypos];

!Variances and residual variances;

xvel yvel xpos ypos;

%C#2%

!Include what is free across classes

!Relationships;

xvel yvel on xpos ypos;

!Intercepts and means;

[xvel yvel xpos ypos];

!Variances and residual variances;

xvel yvel xpos ypos;
